# Effect of polyamines and synthetic polyamine-analogues on the expression of antizyme (AtoC) and its regulatory genes

**DOI:** 10.1186/1471-2091-8-1

**Published:** 2007-01-15

**Authors:** Panagiota S Filippou, Efthimia E Lioliou, Christos A Panagiotidis, Constantinos M Athanassopoulos, Thomas Garnelis, Dionyssios Papaioannou, Dimitrios A Kyriakidis

**Affiliations:** 1 Laboratory of Biochemistry, Department of Chemistry, Aristotle University of Thessaloniki, Thessaloniki, 54124, Greece; 2 Department of Pharmaceutical Sciences, Aristotle University of Thessaloniki, Thessaloniki, 54124, Greece; 3 Laboratory of Synthetic Organic Chemistry, Department of Chemistry, University of Patras, 26500 Patras, Greece; 4 The National Hellenic Research Foundation 48, Vas. Constantinou Ave 11635, Athens, Greece; 5 Present address: Laboratory of Procaryotic Molecular Genetics, Smurfit Institute of Genetics, Trinity College, Dublin, Ireland

## Abstract

**Background:**

In bacteria, the biosynthesis of polyamines is modulated at the level of transcription as well as post-translationally. Antizyme (Az) has long been identified as a non-competitive protein inhibitor of polyamine biosynthesis in *E. coli*. Az was also revealed to be the product of the *atoC *gene. AtoC is the response regulator of the AtoS-AtoC two-component system and it functions as the positive transcriptional regulator of the *atoDAEB *operon genes, encoding enzymes involved in short chain fatty acid metabolism. The antizyme is referred to as AtoC/Az, to indicate its dual function as both a transcriptional and post-translational regulator.

**Results:**

The roles of polyamines on the transcription of *atoS *and *atoC *genes as well as that of *atoDAEB(ato) *operon were studied. Polyamine-mediated induction was tested both in *atoSC *positive and negative *E. coli *backgrounds by using β-galactosidase reporter constructs carrying the appropriate promoters patoDAEB, patoS, patoC. In addition, a selection of synthetic polyamine analogues have been synthesized and tested for their effectiveness in inducing the expression of *atoC/Az*, the product of which plays a pivotal role in the feedback inhibition of putrescine biosynthesis and the transcriptional regulation of the *ato *operon. The effects of these compounds were also determined on the *ato *operon expression. The polyamine analogues were also tested for their effect on the activity of ornithine decarboxylase (ODC), the key enzyme of polyamine biosynthesis and on the growth of polyamine-deficient *E. coli*.

**Conclusion:**

Polyamines, which have been reported to induce the protein levels of AtoC/Az in *E. coli*, act at the transcriptional level, since they cause activation of the *atoC *transcription. In addition, a series of polyamine analogues were studied on the transcription of *atoC *gene and ODC activity.

## Background

Polyamines are indispensable cellular components implicated in many physiological functions, such as DNA replication and repair, transcription, protein synthesis and post-translational protein modifications [1]. Together with magnesium ions, polyamines account for the majority of the intracellular cationic charges [2,3] and they are essential for the normal cell growth and viability of almost all living cells [4,5]. The intracellular polyamine concentrations need to be maintained within relatively narrow limits in order to both ensure optimal cell growth and avoid potential toxic effects arising from the presence of high concentrations of these polycations.

Polyamine homeostasis involves a combination of several sensitive feedback systems regulating their synthesis, degradation and transport [6]. Regulation of polyamine biosynthesis is complex and the key biosynthetic enzyme, ODC is one of the most highly regulated enzymes [7]. The protein levels of ODC and/or its activity are modulated at the transcriptional, translational and post-translational levels [8]. The post-translational regulation of ODC is mainly mediated by polyamine-inducible non-competitive protein inhibitor(s), termed antizymes [9-13]. The mammalian antizyme has also been found to promote the ubiquitin-independent degradation of ODC by the 26S proteasome [14,15], as well as to negatively regulate polyamine transport [16,17].

In *Escherichia coli*, the biosynthesis of polyamines is modulated both at the level of transcription as well as post-translationally [18,19]. The post-translational regulation of polyamine biosynthesis takes place either directly by feedback inhibition of ODC activity by polyamines [20] or indirectly by polyamine-inducible protein inhibitors [2,9].

The *E. coli *antizyme (Az) has been identified as a non-competitive protein inhibitor of ODC, the synthesis of which is induced by polyamines [9,10]. The cloning and sequencing of the *E. coli *Az gene [21] disclosed unexpectedly that Az might also have a second function as the transcriptional regulator of a two-component system (TCS) family [22]. Indeed, it was shown that Az is identical to the gene product of *atoC *[23,24], which is a positive transcriptional regulator of the *atoDAEB *operon genes, encoding enzymes involved in short chain fatty acid metabolism [25,26]. Therefore, the Az is now referred to as AtoC/Az, to indicate its dual function as both a transcriptional and post-translational regulator [27,28].

TCSs are usually composed of an inner membrane sensor histidine kinase and a cognate response regulator, which frequently is a transcriptional activator [22]. Recent work from our laboratory has provided biochemical evidence that AtoS is indeed a membrane-bound sensor histidine kinase that phosphorylates the response regulator AtoC/Az, both constituting a TCS [27,28]. The ability of a recombinant cytosolic region of AtoS to autophosphorylate, albeit at a very low rate, has also been demonstrated in a recent global analysis of *E. coli *TCSs [29]. The *in vitro *trans-phosphorylation of the AtoC/Az by a truncated form of its cognate AtoS kinase, where both proteins were expressed as recombinant his-tagged fusions, has also been demonstrated by our group (unpublished data). Acetoacetate is the only inducer of the AtoS-AtoC TCS identified thus far, for the activation of the AtoS-AtoC TCS. Upon activation, AtoS-catalyzed AtoC phoshorylation is essential for the transcriptional activation of the *atoDAEB *operon, the products of which are essential for the catabolism of short-chain fatty acids [25,26]. Recent global analyses of the *E. coli *TCSs [29,30] have revealed that the AtoS-AtoC TCS might not affect solely *atoDAEB *regulation but it could be involved in a number of additional processes such as flagella synthesis, chemotaxis [29] and sodium but not potassium sensitivity [30]. The cross-regulation between AtoS-AtoC and EnvZ-OmpR TCSs has been also reported, as mutations in the latter TCS affect expression of *atoC *[29]. According to our data, the AtoS-AtoC TCS also acts directly on the *atoDAEB *operon transcription to enhance poly-hydroxy-butyrate (cPHB) biosynthesis in *E. coli *[31].

The Az levels are induced when polyamine levels rise [13], which is expected for a protein which elicits its effects by binding stoichiometrically to ODC. However, the mechanisms for this induction vary in different organisms. The mammalian antizyme levels are mainly regulated at the level of translation by polyamine-inducible programmed +1 ribosomal frameshifting [32], whereas the levels of the *E. coli *antizyme-like proteins S20 and L34 are regulated at the transcriptional level [18,19].

Although the levels of the *E. coli *AtoC/Az have been found to increase upon cell exposure to high polyamine concentrations, there has been no evidence on the molecular basis of this induction. The aim of the present study was to elucidate the mechanism of polyamine-mediated induction of AtoC/Az in *E. coli*.

Polyamine analogues have been developed and used as probes in an effort to clarify the functions of natural polyamines [33,34] as well as potential cancer chemotherapeutic agents and in treating several parasitic diseases [35-39]. Here we used 14 newly synthesized polyamine analogues as tools for monitoring the mechanism(s) by which endogenous polyamines: a) modulate AtoC/Az levels by affecting *atoC *gene transcription, b) affect transcription of other genes that share a topological and/or functional relevance with *atoC*, i.e. the neighboring *atoS *gene, encoding the AtoS kinase of the AtoS-AtoC TCS, and the *atoDAEB *operon which is regulated by AtoC/Az, and c) alter the activity of ODC, the key enzyme for polyamine biosynthesis.

## Results

### Effect of putrescine and spermidine on the transcription of the *atoS-atoC* two component system genes and the *ato* operon

**Figure 1:**
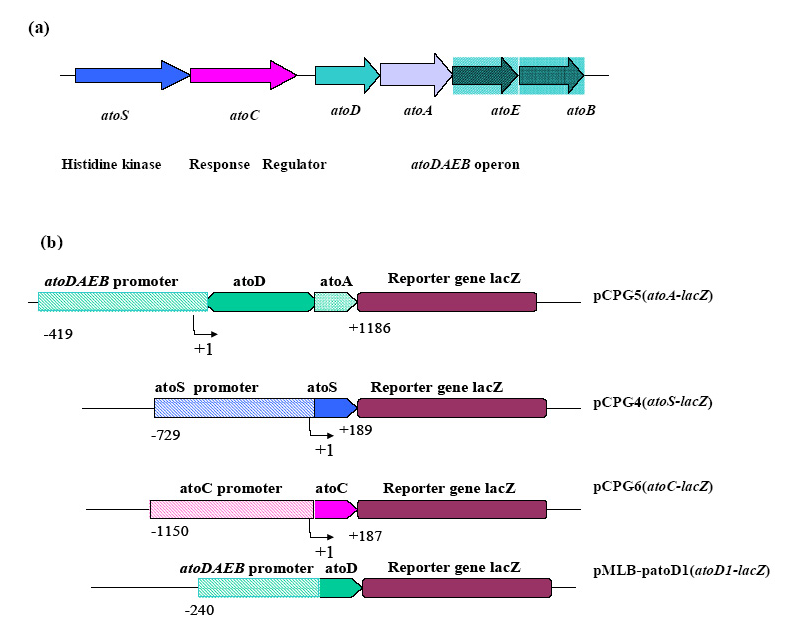
**a. **Structural organization and direction of transcription of *atoSCDAEB *genes in *E. coli*.**b. **The constructs of plasmid pMLB1034 are carrying various promoters fused to a promoterless lacZ gene. pMLB-patoD1(*atoD1-lacZ*) and pCPG5 (*atoA-lacZ*) are carrying the promoter of the *atoDAEB *operon, extending 240 and 460 bp upstream the translational start of the *atoD *gene respectively. pCPG4(*atoS-lacZ*) represents the promoter of *atoS *and a part of the *atoS *gene and pCPG6(*atoC-lacZ*) represents the promoter of *atoC *and a part of the *atoC *gene.

**Figure 2:**
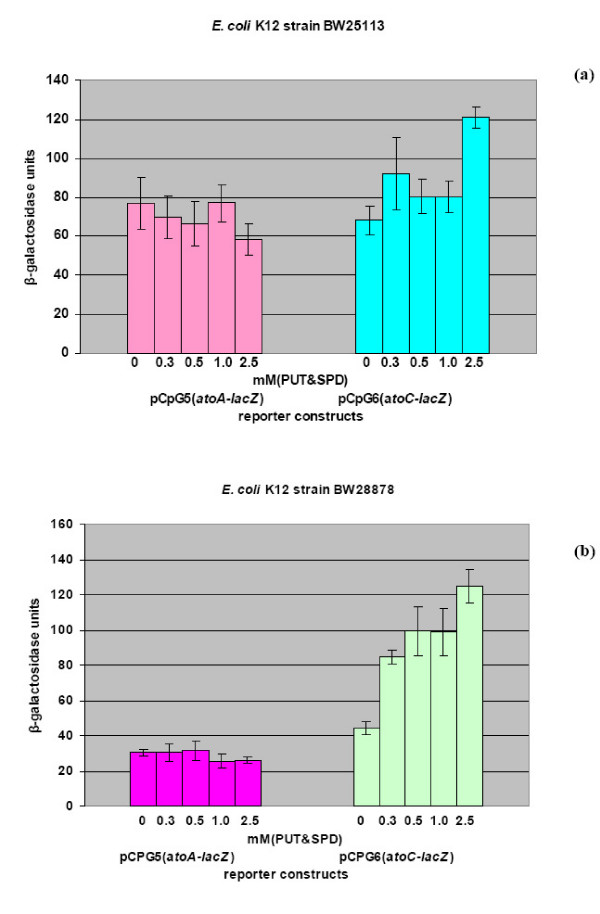
The effect of the polyamines PUT and SPD in the transcription of the *atoSC *two component system genes.**(a)**The *E. coli *K12 strain BW25113 (*atoSC*^+^) and **(b) ***E. coli *K12 strain BW28878(*ΔatoSC*), were transformed with recombinant plasmids, pCPG5(*atoA-lacZ*) and pCPG6(*atoC-lacZ*), carrying the various promoters of the *atoSC *two component system. Polyamine-mediated induction was measured by assaying β-galactosidase expression in the presence of increasing concentrations of polyamines (0, 0.3, 0.5, 1 and 2.5 mM). The results are presented from three independent experiments, while in each experiment two clones from each transformant were tested.

**Figure 3:**
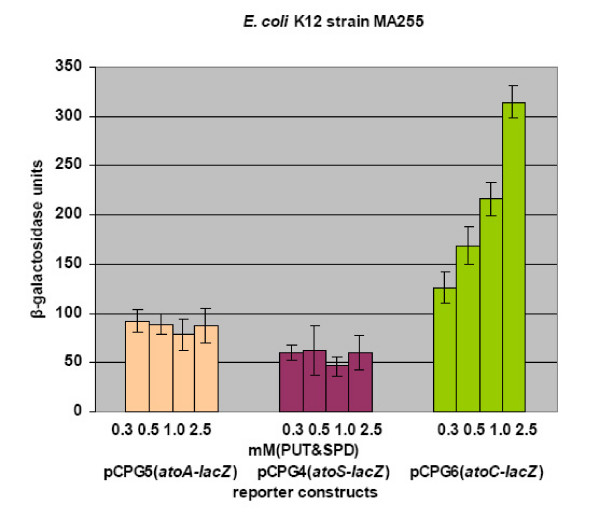
The effect of polyamines in the transcription of the *atoSC *genes in *E. coli *strain MA255. The *E. coli *K12 strain MA255 was transformed with plasmids pCPG4(*atoS-lacZ*), pCPG5(*atoA-lacZ*) and pCPG6(*atoC-lacZ*). Polyamine-mediated induction was measured by assaying β-galactosidase expression in the presence of the indicated concentrations of polyamines (in mM).

The ability of the reporter constructs (Fig 1), carrying *lacZ *fused to either of the promoters of the *atoSC *two component system (i.e the *atoS *or the *atoC *promoter) or to its regulated genes, to respond to polyamines was evaluated in three *E. coli *strains. The isogenic *E. coli *strains, BW25113 and BW28878 that either carry the wild-type *atoSC *(BW25113) [29] or a deletion of the *atoSC *genomic region (BW28878) [30] and the MA255 strain (lacking ODC and AUH) [9], were transformed with the recombinant plasmids described at Fig. 1. Initially, polyamines were added in the growth medium as a mixture of putrescine and spermidine at the final concentrations of 0.3, 0.5, 1.0 and 2.5 mM each. The ability of the reporter constructs to respond to polyamines was determined, in all three *E. coli *strains, by assaying β-galactosidase expression. As shown in Fig. 2 &3, polyamines caused activation of transcription of the *atoC *gene in a polyamine concentration-dependent manner.

The specificity of this effect was demonstrated through the lack of activation upon polyamine addition, of either the *ato *operon promoter, when *atoA-lacZ *(Fig. 2 &3) or *atoD1-lacZ *constructs were used (extending 460 and 240 bp upstream of the translational start of *atoD *gene, respectively), or *atoS *(data not shown) for all three *E. coli *strains tested.

### Effect of the physiological polyamines and diaminopropane on the activation of the *atoC* gene

**Figure 4:**
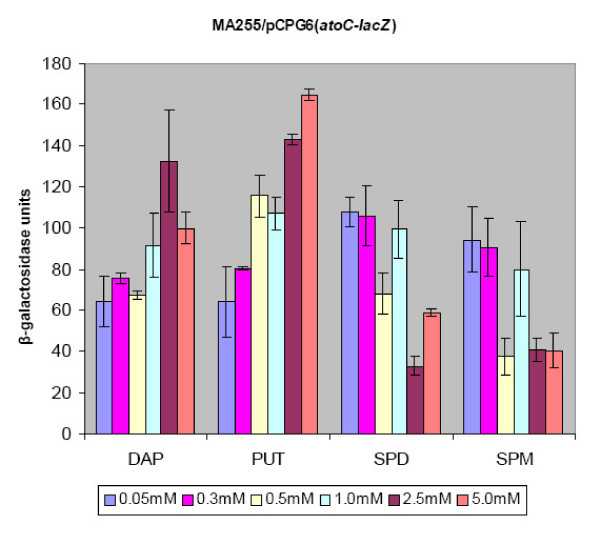
The effect of each polyamine alone PUT, DAP, SPD and SPM in the transcription of the *atoC *gene in *E. coli *strain MA255. Increasing concentrations of each polyamine were added in the growth medium (see Methods) and the transcriptional activity of the reporter construct pCPG6(*atoC-lacZ*) was measured by assaying β-galactosidase activity.

In order to clarify which of the two polyamines played the major role in the transcriptional activation of the *atoC *gene the experiments were repeated in the presence of increasing concentrations of each polyamine. Specifically, *E. coli *MA255 carrying the reporter plasmid pCPG6 (*atoC-lacZ*) were grown in the presence of each of the polyamines diaminopropane, putrescine, spermidine or spermine (0.05, 0.3, 0.5, 1, 2.5 and 5 mM) alone. The results of the β-galactosidase assay suggested that putrescine and diaminopropane elicited a more pronounced effect in the transcriptional activation of the *atoC *gene (Fig. 4). In contrast, spermidine and spermine not only failed to induce *atoC *but they slightly inhibited its expression.

### Effect of polyamine analogues on the transcription of the *atoC* and *ato* operon genes of *E. coli* strain MA255 (*speB*^-^, *speC*^-^) and BW25113 strain

**Figure 5:**
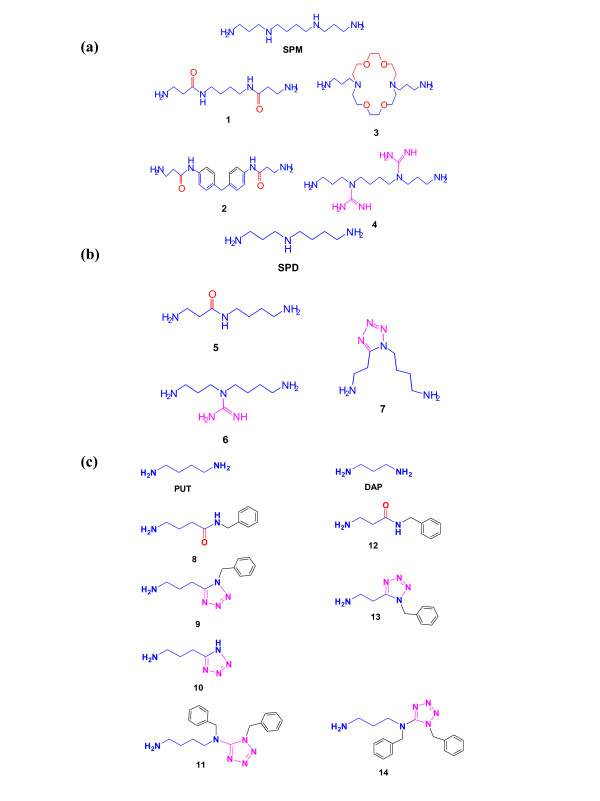
Structures of **a. **SPM, **1–4**, **b**. SPD, **5–7**, **c. **PUT, **8–11 **and DAP, **12–14**, analogues.

A series of polyamine analogues (**1–14**, Fig. 5) has been synthesized and used to compare their biological effects with those of polyamines. These analogues were grouped into three structural types: the spermine analogues (Fig. 5a), the spermidine analogues (Fig. 5b) and the putrescine and diaminopropane analogues (Fig. 5c).

The effect of the polyamine analogues on the growth of a polyamine-dependent *E. coli *strain was investigated. *E. coli *MA255, whose growth depends on the presence of polyamines, were cultivated in media containing polyamine analogues (1 mM) or 0.5 mM putrescine and 0.5 mM spermidine (i.e. 1 mM in total). Following a longer lag period in the presence of the polyamine analogues, the bacteria displayed similar doubling times during the logarithmic phase irrespective of the polyamine or analogue used (data not shown).

The effects of polyamine analogues on *atoC *and *ato *operon promoters were tested using *E. coli *strains MA255 and BW25113 carrying the appropriate reporter constructs, i.e. pCPG6 (*atoC-lacZ*) and pCPG5 (*atoA-lacZ*), and the results are presented in Tables 1 &2. To facilitate comparisons between different sets of data, the β-galactosidase activities measured in the polyamine auxotrophic strain MA255 growing in the presence of 0.3 mM of putrescine and spermidine (Table 1) or in the wild-type strain BW25113 growing in their absence (Table 2) were arbitrarily defined as 100%.

**Table 1:** Effect of polyamine analogues in the transcriptional activation of the *atoC *gene and *atoDAEB *operon in *E. coli *strain MA255.

**SUBSTANCES**	**% β-galactosidase activity MA255/(*atoC-lacZ*)**	**% β-galactosidase activity MA255/(*atoA-lacZ*)**
Control (Putrescine & spermidine/0.3 mM each)	100%	100%
***1 mM concentration***		
***SPERMINE ANALOGUES***		
**1**	67 ± 4.86	200 ± 10.66
**2**	142 ± 8.69	181 ± 2.09
**3**	135 ± 4.69	177 ± 1.39
**4**	43 ± 3.11	38 ± 2.38
***SPERMIDINE ANALOGUES***		
**5**	78 ± 2.56	155 ± 9.12
**6**	179 ± 10.9	114 ± 3.66
**7**	110 ± 8.50	208 ± 8.80
***PUTRESCINE ANALOGUES***		
**8**	108 ± 10.93	59 ± 5.74
**9**	119 ± 7.34	43 ± 8.80
**10**	124 ± 10.30	64 ± 10.66
**11**	172 ± 6.42	45 ± 5.85
***DIAMINOPROPANE ANALOGUES***		
**12**	248 ± 8.02	69 ± 8.14
**13**	169 ± 8.66	111 ± 10.24
**14**	452 ± 2.17	218 ± 6.77

The data are presented as % activity of β-galactosidase for MA255 cells grown in the presence of 0.3 mM putrescine and spermidine and 1 mM of the indicated polyamine analogue. The values represent the means ± SD from two separate experiments.

**Table 2:** Effect of polyamine analogues in the transcriptional activation of the *atoC *gene and *atoDAEB *operon in *E. coli *strain BW25113.

**SUBSTANCES**	**% β-galactosidase activity BW25113/(atoC-lacZ)**	**% β-galactosidase activity BW25113/(atoA-lacZ)**
Control (No polyamines added)	100%	100%
***1 mM concentration***		
***SPERMINE ANALOGUES***		
***spermine***	98 ± 3.38	88 ± 1.98
**1**	98 ± 1.9	80 ± 5.96
**2**	156 ± 1.62	79 ± 1.59
**3**	80 ± 1.41	58 ± 2.49
**4**	N.D	N.D
***SPERMIDINE ANALOGUES***		
***spermidine***	105 ± 1.38	107 ± 3.37
**5**	107 ± 1.82	60 ± 2.84
**6**	128 ± 1.73	52 ± 3.76
**7**	N.D	N.D
***PUTRESCINE ANALOGUES***		
***putrescine***	170 ± 4.29	110 ± 3.28
**8**	125 ± 1.63	76 ± 1.93
**9**	N.D	N.D
**10**	216 ± 3.92	233 ± 6.30
**11**	N.D	N.D
***DIAMINOPROPANE ANALOGUES***		
***diaminopropane***	200 ± 4.19	87 ± 2.26
**12**	238 ± 6.44	128 ± 5.47
**13**	185 ± 9.81	202 ± 9.68
**14**	303 ± 6.80	193 ± 7.35

Data are presented as % activity of β-galactosidase for BW25113 cells grown in the absence of polyamines or in the presence of the indicated polyamine or polyamine analogue. The values represent the means ± SD from two separate experiments.

It was found that some of these analogues activated the transcription of the reporter genes more than their parent polyamines. In general, the most potent transcriptional activators (compounds **10**, **12 **and **14**) were putrescine and diaminopropane (DAP) analogues. Comparison of these two types of analogues (e.g. **8 **against **12**, **9 **against **13 **and **11 **against **14**), revealed that the most active compounds were clearly the DAP analogues. Compound **14 **was the most potent activator of both *atoDAEB *and *atoC *promoters. The effects appeared to be specific since some of the analogues showed different action on the two promoters since they stimulated, at least to a certain extend *atoC *while they repressed the *atoDAEB *(*ato*) operon and vice versa. It is worth noticing that amongst spermine analogues, compound **4**, bearing the strongly basic guanidine group on atoms N-4 and N-9 of the SPM backbone repressed the synthesis of both *atoC *and *atoDAEB *operon.

**Figure 6:**
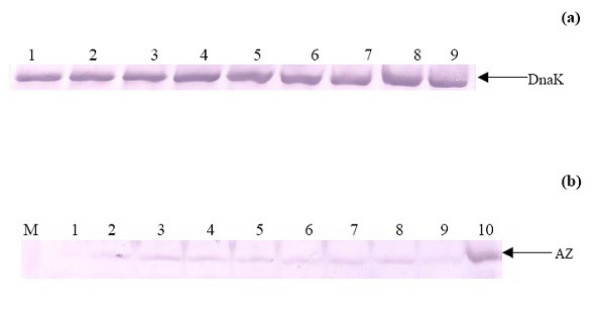
**a **Effect of polyamines or polyamine analogues on endogenous DnaK expression. Western blot of total cell extracts of *E. coli *K12 cells BW25113 (*atoSC*^+^) grown to an OD_600 _0.8–0.9 (15 μl), was performed to detect the expression of the heat shock protein DnaK. Lane 1: BW25113 cells with no inducer. Lanes 2–4: BW25113 cells treated with PUT, SPD, or SPM, respectively. Lanes 5–9: BW25113 cells treated with 1 mM polyamine analogue **2, 10, 12, 13, 14**, respectively. **6 b. **Effect of polyamines or polyamine analogues on endogenous AtoC/Az expression. The *E. coli *K12 cells BW25113(*atoSC*^+^) were cultured in the presence of 1 mM of the indicated polyamine analogue or each polyamine, respectively. Total cell extracts of each culture grown to an OD_600 _0.8–0.9 (20 μl) were analyzed. The immunoblot analysis was performed using the purified polyclonal rabbit antibody of AtoC/Az. BW28878 cells, not expressing the atoC/Az protein, were used as negative control. The high copy plasmid pUC-Az [21], overexpressing the AtoC/Az protein, was used as a positive control. Lane 1: BW28878 cells, Lane 2: BW25113 cells with no inducer Lanes 3–5: BW25113 cells treated with PUT, SPD or SPM, respectively. Lanes 6–9: BW25113 cells treated with analogues **10**, **12**, **14**, **13**. Lane 10: BW25113 cells carrying the pUC-Az plasmid.

To determine whether the above effects on gene expression were specific, or whether they resulted from polyamine analogue-induced stress effects on the cells exposed to them, we measured the levels of the heat shock protein DnaK using immunoblot analysis. The absence of an increase in the DnaK levels in cells exposed to polyamine analogues indicated that they did not induce the heat-shock response (Fig. 6a).

### Polyamine and polyamine analogues effect on AtoC/Az protein levels

Since polyamines and polyamine analogues activated the transcription of *atoC/Az*, it was investigated whether this activation also leads to AtoC/Az protein accumulation in the cell, thus affecting the translational mechanism.

Therefore, BW25113 cells were exposed to polyamines or their analogues, at the final concentration of 1 mM, grown to an OD_600 _0.8–0.9 and cell extracts were prepared as described for immunoblot analysis (Fig. 6b).

These experiments showed that AtoC/Az protein levels are induced by polyamines (lanes 3–5) and the most active polyamine analogues (**12**, **14**, lanes 6, 8). This induction is clear despite the very low intracellular levels of AtoC/Az, which was barely detectable in total extracts from cells growing in the absence of polyamines (lane 2). Extracts from cells lacking the AtoC/Az protein (BW28878) were used as negative control and BW25113 cells transformed with plasmid pUC-Az [21] overexpressing AtoC/Az were used as a positive control for the blot.

### Effect of polyamine analogues on ODC activity and protein levels

It has been demonstrated that polyamines cause a decrease in the *E. coli *ODC mRNA and protein levels and that the specific activity of ODC decreases significantly more than its protein levels, due to its post-translational inhibition by antizyme(s) [9,18,19]. Considering that the polyamine analogues, like polyamines can affect gene expression, their effects on ODC activity were also investigated.

Thus, the ODC activity was assayed *in vitro *using extracts from polyamine analogue-exposed *E. coli *cells. These extracts were prepared from cells grown in the mineral medium M9 to OD_600 _0.8–0.9 and the assays were performed as described in Methods. Provided that some polyamine analogues behaved like natural polyamines, it was expected that the specific activity of ODC would decrease as the levels of AtoC/antizyme, its post-translational inhibitor, rise [19].

Table 3 shows that indeed *E. coli *exposure to some of the polyamine analogues decreased the ODC specific activity. It is interesting that the polyamine analogue 14, which has the highest negative impact on ODC specific activity (Table 3) was also found to be the most potent activator of *atoC *expression (Tables 1 &2).

**Table 3:** Effect of polyamine analogues on ODC activity.

**BW25113 strain/1 mM Substance**	**% ODC activity**	**% Inhibition of ODC activity**	**ODC Units/10 μl**	**Specific ODC activity (Units/mg protein)**
**Control**	100	0	4.79	239.5 ± 3.5
**PUT**	40.66	59.33	1.95	84.78 ± 0.5
**SPD**	47.89	52.11	2.29	76.33 ± 0.2
**SPM**	76.94	23.06	3.68	153.33 ± 2.5
**8**	74.98	25.02	3.59	211.17 ± 4.0
**10**	51.78	48.22	2.48	118.09 ± 0.3
**12**	37.52	62.48	1.79	99.44 ± 1.5
**13**	61.36	38.64	2.94	108.88 ± 2.3
**14**	56.82	43.18	2.72	82.42 ± 0.2

*E. coli *BW25113 was grown to an OD_600 _0.8–0.9 in M9 medium in the absence (control) or in the presence of 1 mM polyamine (PUT, SPD, SPM) or 1 mM polyamine analogue. ODC activity and protein levels were determined as described in Materials and Methods. Specific activities are expressed as μmol ^14^CO_2 _liberated/mg enzyme protein/h of incubation. The values represent the means ± SD from two separate experiments.

**Figure 7:**
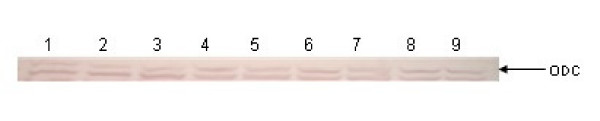
Effect of polyamine analogues on endogenous ODC expression. *E. coli *K12 cells BW25113 (*atoSC*^+^) were grown in the presence of 1 mM of the indicated polyamine or polyamine analogue, to a cell density of 0.8–0.9. The total cell extracts from each culture (20 μl) were subjected to SDS-PAGE 10% and immunostained with anti-ODC. Lane 1: BW25113 cells, with no inducer added. Lanes 2–4: BW25113 cells, treated with 1 mM PUT or SPD or SPM. Lanes 5–9: BW25113 cells treated with 1 mM of each polyamine analogue **8, 10**, **12**, **13 **and **14**, respectively.

Since growth of *E. coli *cells in the presence of polyamines results in decreased ODC protein levels [19], it was investigated whether the reduced ODC activity in extracts from polyamine analogue-exposed cells (Table 3) similarly resulted from such a reduction. Immunoblot analysis, with a rabbit polyclonal anti-ODC antibody, was performed to measure the levels of ODC protein in the same extracts as those used for the in vitro ODC assays. These experiments indicated that, despite their negative effects on ODC activity, the polyamine analogues caused no reduction on the ODC protein levels (Fig 7.)

## Discussion

Polyamines are essential cellular components for the growth and proliferation of all living cells. Regulation of polyamine biosynthesis is complex and the key biosynthetic enzyme ODC, is one of the most highly regulated enzymes [7], the levels and/or activity of which are modulated at the transcriptional, translational and post-translational levels [8].

Antizyme is long known as a polyamine-inducible, non-competitive inhibitor of ODC [9,13-15]. Az has also been found to be the product of *atoC *gene whose product is the response regulator of the AtoS-AtoC two component system. Recent work from our laboratory has shown that AtoS and AtoC indeed constitute a two component system and that they participate in *atoDAEB *operon expression upon acetoacetate induction [27,28].

*E. coli *Az is induced by polyamines and inhibits the activity of ODC by forming an Az-ODC complex [9]. Little is known, however, about the mechanism of this induction, in contrast to the eukaryotic antizyme, which is induced by polyamines at the translational level [27,32].

In this work we attempted to elucidate the way by which polyamines induce AtoC/Az in *E. coli *and furthermore to clarify their effect in the transcription of other genes that share a topological and/or functional relevance with *atoC/Az*. The measurement of the β-galactosidase activity in cells carrying various promoter-*laZ *constructs indicated that *atoC*, but no other gene tested, expression was activated upon growth in increasing polyamine concentrations. This activation in *atoC *expression was not strain-specific since it was manifested in all three *E. coli *strains studied.

The combination of both putrescine and spermidine was not required since *atoC *expression was found to be induced by each polyamine alone (Fig. 4). The data derived from both *E. coli *MA255 (Fig. 4) and BW25113 strains (Table 2) indicated that PUT and the non-natural diamine DAP were the main transcriptional effectors. The *in vivo *transcriptional effects of DAP and PUT suggested that a distance of three to four carbon atoms between the two amino groups had the maximal effect on the transcriptional activation of the *atoC*. The specificity of PUT and DAP to exert this effect was strengthened by the inability of the multivalent SPD or SPM to activate the *atoC *gene.

Subsequently 14 polyamine analogues were synthesized and tested both for their ability to support the growth of a polyamine-dependent *E. coli *strain and to affect an essential biological process, such as the transcriptional activation of genes of *atoDAEB *operon.

Most of these analogues, at the final concentration of 1 mM, were used by *E. coli *strain MA255 as carbon sources and supported cell growth. The analogues demonstrated a differential effect on gene expression as evidenced by the facts that: i) a number of analogues activated the *atoC *transcription, without affecting the transcription of the *ato *operon (compound **12**) and vice versa (compounds 1&7) and ii) some of the analogues seemed to activate both the *atoDAEB *(*atoA-lacZ*) and the *atoC *transcription (*atoC-lacZ*) (compounds **10, 14**, Table 2.). These results could not attributed to a broad effect of these compounds, since the levels of the heat shock protein DnaK were not affected, as shown by immunoblot analysis of total extracts of cells exposed to these analogues (Fig. 6a).

Analysis of the effects of the different analogue types on *atoC *gene expression (e.g. **8 **against **12**, **9 **against **13 **and **11 **against **14**), revealed that the most active compounds were the DAP analogues, with compound 14 being the most potent. These data established the significance of charge distribution and chain flexibility on polyamine mode of action in *atoC *transcription.

In the cases where both *atoC *and *atoDAED *were activated by the analogues, the activation of *atoDAEB *could not be attributed to the enhanced intracellular accumulation of AtoC/Az, the product of *atoC*, but rather to their direct effects on *atoDAEB *transcription. This is surprising since none of the physiological polyamines produced an analogous effect.

The regulatory role of Az in the feed-back inhibition of ODC, the rate limiting enzyme in polyamine biosynthesis, was investigated by the ability of the PUT and DAP synthetic analogues to reduce the ODC activity in *E. coli*. The inhibition of ODC activity by these analogues (Table 3) could result from negative transcriptional effects of the polyamine analogues on *speC*, the gene that encodes ODC, or/and indirectly through antizyme induction, the intracellular accumulation of which would increase the proportion of ODC-Az inactive complex. The latter possibility was more probable in view of the fact that several polyamine analogues activated the expression of *atoC*, which encodes antizyme. Indeed, immunoblot analysis indicated that the analogues that activated *atoC *transcription also produced increased Az accumulation, without affecting ODC protein levels (Fig. 6b and 7).

Interestingly, elevated levels of ODC have been associated with highly proliferating cells [2,3,5,7,13,33] and polyamine analogues as well as drugs that influence intracellular polyamine levels have shown antiproliferative activity [35-38]. Despite the differences between bacterial and eukaryotic mechanisms that govern Az induction and regulation of polyamine biosynthesis, substances that act as inducers of Az could reduce polyamine levels and provide a lead for the development of agents capable of cell growth arrest.

## Conclusion

Polyamines, which have been reported to induce AtoC/Az in *E. coli*, activated the expression of *atoC*, the gene that encodes AtoC/Az. A series of synthetic polyamine analogues have been tested for their effectiveness on the expression of the *atoC*, as well as that of the *atoDAEB *(*ato*) operon. Putrescine and diaminopropane analogues, activated *atoC *transcription, indicative of the structural requirements of diamines for Az induction. In addition, this *atoC *induction resulted in accumulation of Az protein and inhibition of ODC activity. This inhibition is likely due to the formation of inactive ODC-Az complexes, since in bacteria grown in the presence of polyamine analogues did not affect the ODC protein levels.

## Methods

### Bacterial strains, plasmids and culture conditions

#### Bacterial strains

*E. coli *K-12 strains were transformed with the appropriate plasmids. *E. coli *strain MA255 [F^-^, *thr*^-^, *leuB6*(Am), *fhuA2*?, *lacY1*, *glnV44*(AS)?, *gal*-*6*, λ^-^, *relA1*?, *CanR*?-1, *speB*, *speC*, *rpsL133*(strR), *χylA7*, *mtlA2*, *thi*^-^] was obtained from *E. coli *genetic stock Center, Department of MCDB, Yale university.

*E. coli *K-12 BW25113 [*lacI*^*q *^*rrnB3 *Δ*lacZ4787 hsdR514 *DE*(araBAD)567 *DE*(rhaBAD)568 rph-1*] and BW28878 [BW25113, lacking AtoS-AtoC system, Δ*atoSC*] were a kind gift from Dr Hirofumi Aiba (Laboratory of Molecular Microbiology, School of Agriculture, Nagoya University, Japan).

#### Plasmids

The genetic organisation of the *ato *locus is shown in Figure 1a. The plasmids used in this study are carrying various promoters of genes, fused to a promoterless *lacZ *gene on pMLB1034 vector. These reporter constructs, carrying the promoters of the *ato *operon and the regulatory *atoS-atoC *two component system genes, can direct the synthesis of the *lacZ *gene under the control of their cognate promoters. pCPG4 (*atoS-lacZ*), pMLB-patoD1(*atoD1-lacZ*), pCPG5 (*atoA-lacZ*) and pCPG6 (*atoC-lacZ*), are carrying the *atoS *promoter, various lengths of the *atoDAEB *promoter (extending 240 and 460 bp upstream the translational start of the atoD gene, respectively) and the *atoC *promoter (Fig 1b)

#### Culture conditions

*E. coli *cells were grown at 37°C with vigorous shaking in M_9 _minimal medium [40], supplemented with 0.1 mM CaCl_2_, 1 mM MgSO_4_, 0.5% w/v glucose, 1.7 μM FeSO_4, _1 μg/ml thiamine, 50 μg/ml DL-leucine and threonine (for the growth of MA255) and 80 μg/ml DL-proline (for BW25113 and BW28878 strains). Ampicillin was used at the final concentration of 100 μg/ml.

*E. coli *strain MA255 lacking the biosynthetic enzymes ODC and agmatine ureohydrolase (AUH), is unable to synthesize polyamines and grows only in the presence of exogenously supplied polyamines. Therefore, polyamines (PUT and SPD) were also added in the growth medium at the final concentration of 0.05 mM which was the lowest concentration necessary for growth.

The strains to be tested were grown overnight, then diluted 10-fold in the growth medium containing polyamines or polyamine analogues and the cells were grown to OD_600 _0.4–0.7. The cells were harvested by centrifugation at 8000 × g and washed twice with M_9 _before the β-galactosidase assay.

For the *in vitro *ODC activity assay, *E. coli *cells were grown in M_9 _mineral medium, in the absence or presence of 1 mM polyamines or polyamine analogues. The cells were harvested when the cultures reached late logarithmic phase OD_600 _0.8–0.9 by centrifugation at 8000 × g for 10 min. The cell pellets were washed twice with ice-cold buffer saline and stored at -20°C until use.

#### β-Galactosidase assay

β-Galactosidase activity assays were performed using the the *E. coli *MA255 (*speC*^-^*, speB*^-^), BW25113 (*atoSC*^+^) and BW28878(*ΔatoSC*), carrying the appropriate plasmids, as described previously [40]. In all experiments, strains carrying plasmid pMLB1034 were used as negative controls.

#### Immunoblot analysis

An aliquot of 1 ml of each culture prepared as decribed for the β-galactosidase assays or ODC activity assays, was centrifuged and resuspended in SDS loading buffer of 1.5X (75 mM Tris-HCl pH 6.8, 3% w/v SDS, 0.15% w/v bromophenol blue, 15% v/v glycerol and β-mercaptoethanol at a final concentration of 10% v/v), boiled for 5 minutes and kept at -20°C for immunoblot analysis.

#### ODC activity assay

The cell pellets were suspended in 0.5 ml ice-cold ODC assay buffer (50 mM Tris-HCl pH 8.2, 0.1 mM EDTA, 50 μM 5'-phosphate pyridoxal, 5 mM dithiothreitol), disrupted by sonication in an ice bath for 12 min and centrifuged at 10,000 × g for 10 min at 4°C. Enzyme activity of ODC was assayed in the supernatant of each culture. 10 μl of each supernatant, was incubated with 35 μl ODC assay buffer and 5 μl DL-[1-^14^C]ornithine 0.028 μmol, for 1 h at 37°C. The reaction was terminated with the addition of 0.2 ml of 10% trichloroacetic acid. ODC activity was measured from the liberation of ^14^CO_2 _from the DL-[1-^14^C]ornithine, which was captured on filter paper that was pre-treated with 25 μl soluene-350 and air dried. Filter paper was placed in liquid scintillation fluid. Radioactivity was counted as DPM and expressed in units, as it was defined [9]. The remainder of supernatant was analyzed for protein concentration using Bradford method [41].

### Synthesis of polyamine analogues

#### Spermine analogues 1–4

These compounds were obtained, as the corresponding di-(**1**,**2**) and tetra-(**3**,**4**) trifluoroacetate salts, through routine trifluoroacetic acid (TFA)-mediated deprotection of the corresponding fully protected, with the trityl (primary amino functions) and the *tert*-butoxycarbonyl (guanidine functions) protecting groups, precursors. The precursors to compounds **1 **and **3 **and **2 **and **4 **were obtained according to reported procedures [42,43].

#### Spermidine analogues 5–7

These compounds were obtained, as the corresponding di-(**5**,**7**) and tri-(**6**) trifluoroacetate salts, through routine trifluoroacetic acid(TFA)-mediated deprotection of the corresponding fully protected, with the trityl (primary amino functions) and the *tert*-butoxycarbonyl (guanidine function) protecting groups, precursors (Fig. 5b). The precursors to compounds **5 **and **6 **[42-44] and to the compound **7 **[45] were obtained according to reported procedures.

#### Putrescine analogues 8–14

Compounds **8**, **9**, **12 **and **13 **were obtained, as the corresponding trifluoroacetate salts, through routine trifluoroacetic acid (TFA)-mediated deprotection of the corresponding protected, with the trityl (primary amino function) protecting group, precursors (fig. 5c). The precursors to compounds **8**, **9**, **12 **and **13 **were prepared according to reported procedure [45]. Compound **10 **was obtained from the *N*-tritylated compound **9**, through catalytic hydrogenolysis [45]. Finally, compounds **11 **and **14 **were prepared, as the corresponding trifluoroacetate salts, through routine trifluoroacetic acid (TFA)-mediated deprotection of the corresponding protected, with the trityl (primary amino function) protecting group, precursors. The latter were obtained according to a recently developed protocol in our laboratory for the selective *N*-tetrazolation of polyamines.

#### Antibody purification

The rabbit polyclonal antibodies against *E. coli *ODC, Az and DnaK used in this study have been described [19,28,48]. The anti-Az antibody was negatively purified against cross-linked total protein extracts from the Az^- ^*E. coli *strain BW28878. *E. coli *cells BW28878 were grown in Luria-Bertani (LB) at 37°C to an OD_600 _1.5, were harvested and washed twice with saline. Cells were suspended in ice-cold lysis buffer (50 mM NaH_2_PO_4_, 300 mM NaCl) and lysed by lysozyme treatment (1 mg. ml^-1^), for 60 min at 4°C, and sonication. The cell lysate was centrifuged at 9,000 × g (SS-34 rotor) for 20 min at 4°C to remove cell debris and unbroken cells and the supernatant was treated with DNAse I (3 units. ml^-1^) and RNAse A (10 μg. ml^-1^) for 30 min at 4°C. The lysate was further centrifuged at 9,000 × g (SS-34 rotor) for 20 min at 4°C. The supernatant was stirred with formaldehyde 1% at 4°C for 1 hour. 0.2 M Glycine was then added to react with the remaining formaldehyde, for 30 minutes. The cross-linking of proteins by formaldehyde led to the formation of a white protein complex. The protein pellet was centrifuged at 8000 × g for 15 min, washed three times with 5 ml PBS-Glycine 0.2 M, pH 8.0 and incubated overnight at 4°C with the 400μl serum of the rabbit polyclonal antibody of antizyme. The protein pellet of BW28878 cells could absorb all the antibodies except from that of Az because of the lack of the AtoSC proteins. The serum was centrifuged and the supernatant is the purified antibody used for the immunoblot analysis in this study.

#### Electrophoresis and immunoblotting

SDS-polyacrylamide gel electrophoresis (SDS-PAGE) was performed using 10%(w/v) polyacrylamide gels, as described by Laemmli et al [46]. Proteins were transferred to immobilon PVDF membranes following the method of Towbin et al. [47] and immunostained with the rabbit polyclonal antibodies against, ODC, Az and DnaK, respectively, prepared as described [19,28,48].

## Abbreviations

ODC, ornithine dacarboxylase; AUH, agmatine ureohydrolase; Az, antizyme; TCS, two component system; PUT, putrescine; SPD, spermidine; SPM, spermine; DAP, 1,3-diaminopropane.

## Authors' contributions

All authors contributed equally in this paper. All authors read and approved the final manuscript.
